# Mediating effects of self-esteem and self-compassion on the relationship between body dissatisfaction and depression among adolescents with polycystic ovary syndrome

**DOI:** 10.3389/fpubh.2024.1420532

**Published:** 2024-07-25

**Authors:** Huihui Huangfu, Li Li, Wen Shuai

**Affiliations:** ^1^School of Elderly Care Services and Management, Nanjing University of Chinese Medicine, Nanjing, Jiangsu, China; ^2^Institute of Healthy Yangtze River Delta, School of International and Public Affairs, Shanghai Jiao Tong University, Shanghai, China; ^3^Health Management Center, Shanghai First Maternity and Infant Hospital, Shanghai, China

**Keywords:** PCOS, body dissatisfaction, depression, self-esteem, self-compassion, mediation analysis

## Abstract

**Introduction:**

Body dissatisfaction significantly impacts depression among adolescents with polycystic ovary syndrome (PCOS). This relationship is compounded by various factors. Our study aims to explore the roles of self-esteem and self-compassion in the relationship between body dissatisfaction and depression in adolescent with PCOS.

**Methods:**

A cross-sectional study was conducted at the Shanghai First Maternity and Infant Hospital, involving 287 adolescents diagnosed with PCOS from January 2020 to December 2021. Participants completed validated questionnaires covering body dissatisfaction, self-esteem, self-compassion and depression. We utilized correlation and mediation analyses to examine the relationships and mediating effects among these variables.

**Results:**

Body dissatisfaction had a significant positive effect on depression (β = 4.254, *p* < 0.001). Conversely, self-esteem (β = −0.944, *p* < 0.001) and self-compassion (β = −0.318, *p* < 0.001) were negative predictors of depression. Both self-esteem [β = 3.405, 95% CI = (0.151, 0.305)] and self-compassion [β = 1.525, 95% CI = (0.045, 0.165)] were shown to partially mediate the relationship between body dissatisfaction and depression, explaining 37.07% and 16.61% of the total effect, respectively.

**Conclusion:**

This study highlights the importance of fostering self-esteem and self-compassion among adolescents with PCOS to buffer the depressive effects of body dissatisfaction. Interventions aimed at promoting accurate and positive body perceptions, enhancing self-esteem, fostering a supportive attitude toward personal challenges, and maintaining positive emotional states are recommended to decrease the incidence of depression.

## 1 Introduction

Adolescent depression is a significant global concern, negatively affecting individuals and leading to severe psychological and behavioral issues such as low mood, eating disorders, and suicidal tendencies (1). In the last two decades, there has been a noticeable increase in the prevalence of depressive symptoms among adolescents (2, 3), with approximately 14% of adolescents globally experiencing mental health conditions (1). In China, a national mental health study revealed that nearly 25% of adolescents reported experiencing mild to severe depression (4).

A considerable amount of research has focused on the factors and mechanisms underlying depression. Numerous studies have identified a strong link between increased depression risk and body dissatisfaction (5, 6). Body dissatisfaction arises from negative feelings toward one's physical appearance, driven by a discrepancy between one's ideal and actual appearance (7). This issue is particularly pronounced among adolescent girls, where substantial physical changes during puberty can escalate depressive symptoms (8). Polycystic ovary syndrome (PCOS), a prevalent gynecological endocrine disorder affecting adolescent girls, involves body changes like obesity, acne, hirsutism, and acanthosis nigricans that can negatively impact female identity and contribute to body dissatisfaction (9). Although PCOS typically develops during puberty, it can manifest before or after this period, significantly affecting women's physical and mental health and overall quality of life (10). Studies show that PCOS patients exhibit higher levels of body dissatisfaction and depression compared to their healthy peers (11–13), making body dissatisfaction an important depression risk factor among adolescents with PCOS (9).

However, it is crucial to note that not all adolescents with PCOS who experience body dissatisfaction will necessarily develop depression. The relationship between the two may be moderated by subjective cognitive factors like self-esteem and self-compassion (14, 15). Self-compassion, a strategy for emotional regulation, involves adopting a tolerant and kind attitude toward oneself during distress or failure and handling negative events with equanimity (16). Studies suggest that adolescent girls with lower self-compassion levels are more likely to experience negative body dissatisfaction and depressive emotions (17, 18). Self-esteem, defined by Rosenberg (19), refers to the subjective evaluation of one's own worth and the belief in being valued. It has been shown that negative body image during adolescence significantly influences self-esteem (20), and low self-esteem increases the risk of depressive emotions (21).

Although individual impacts of these factors have been extensively studied—as evidenced by research from Bornioli et al. (22) on body dissatisfaction and depression, Castilho et al. (23) on the link between self-compassion and body satisfaction and Takahashi et al. (24) on the relationship between self-compassion and depression—there is a lack of research exploring their interplay. Stapleton et al. (25) studied the mediating role of self-compassion in self-esteem and body image relationships, while Azizi Kutenaee et al. (14) used a structural equation model to examine the direct and indirect relations among clinical PCOS features, depression, self-esteem, and body image. Despite these contributions, there remains a gap in research regarding the role of self-esteem and self-compassion in the relationship between body dissatisfaction and depression among adolescents with PCOS. This study aimed to address this gap by examining the interrelations among these factors and providing empirical evidence to support the prevention and intervention of depression in adolescents with PCOS.

## 2 Materials and methods

### 2.1 Study design

**Figure 1:**
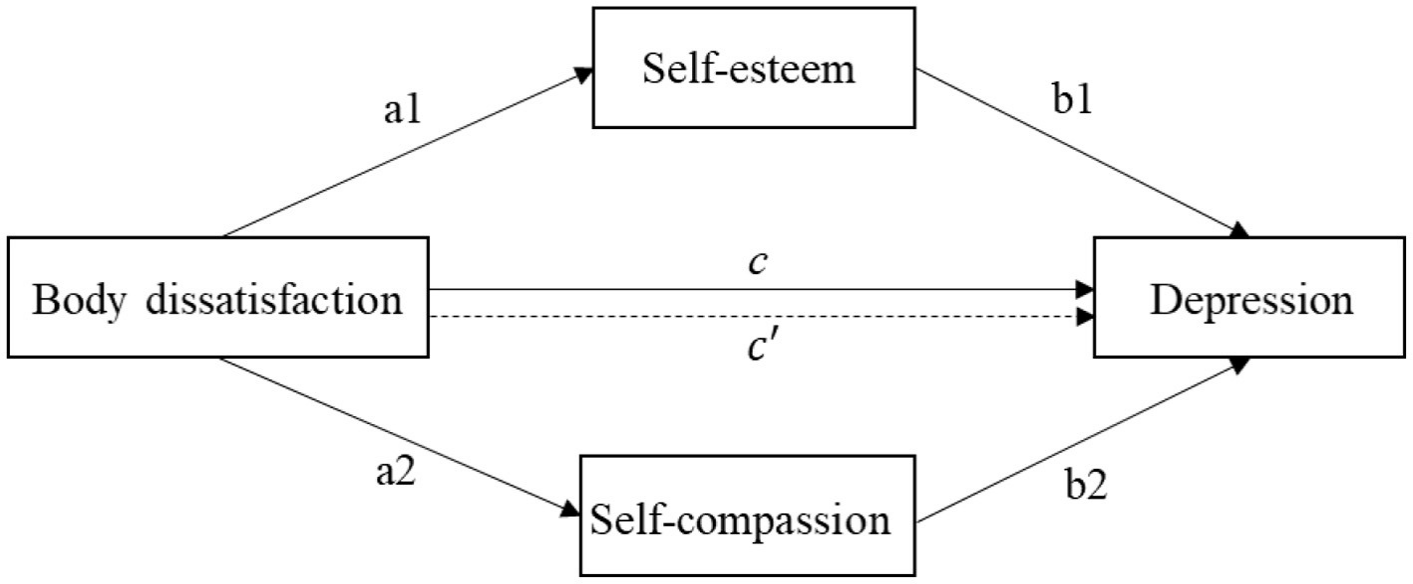
Mediation modeling of body dissatisfaction on depression. a1 and b1 are indirect effect 1, a2 and b2 are indirect effect 2, *c*′ is direct effect, c is total effect.

We used a mediation analysis to examine the roles of self-esteem and self-compassion in mediating the relationship between body dissatisfaction and depression in adolescents with PCOS. Based on existing literature, we developed the following hypotheses to guide our investigation (Figure 1):

Hypothesis 1 (H1): Body dissatisfaction is positively associated with depression.
Hypothesis 2 (H2): Self-esteem acts as a mediator in the relationship between body dissatisfaction and depression.
Hypothesis 3 (H3): Self-compassion serves as a mediator in the relationship between body dissatisfaction and depression.

### 2.2 Measurements

#### 2.2.1 Body dissatisfaction

Body dissatisfaction was assessed using the Negative Physical Self Scale, devised by Chen (26). This scale features 54 items that span various aspects of physical self-perception, including overall appearance, fatness, shortness, facial looks, and thinness. Participants rate their agreement with each item on a 5-point scale from 0 (not at all like me) to 4 (very much like me). Sample items include, “I am very distressed when I think about my weight,” “If it is possible, I will change the way my face looks.” The scale is reliable, with a test-retest reliability coefficient of 0.89 over a 3-week period, and a Cronbach's alpha of 0.86, indicating strong internal consistency. For this study, we focused on three specific dimensions relevant to PCOS symptoms—overall appearance (6 items), facial looks (12 items), and fatness (12 items). A score average below 2 indicates lower dissatisfaction, whereas a score of 2 or above indicates higher dissatisfaction, with higher scores representing greater body dissatisfaction.

#### 2.2.2 Depression

Depression was measured using the Center for Epidemiologic Studies Depression Scale (CES-D), developed by Radloff (27, 28). This 20-item self-report scale assesses the frequency of depressive symptoms over the past week, including restless sleep and feelings of loneliness, with responses ranging from 0 (rarely) to 3 (most or almost all the time). Scores range from 0 to 60, where higher scores indicate more frequent depressive symptoms. The scale includes a cutoff score of 16 to identify individuals at risk for depression and is noted for its high internal consistency and adequate test-retest reliability. It is applicable across various age groups (29).

#### 2.2.3 Self-esteem

Self-esteem was evaluated using the shortened version of Rosenberg's Self-Esteem Scale (SES) from 1965 (19). This 10-item scale measures self-worth with items rated from 1 (strongly disagree) to 4 (strongly agree), where higher scores reflect higher self-esteem. The SES is particularly noted for its high internal consistency, with a Guttman scale coefficient of reproducibility of 0.92 and test-retest reliabilities of 0.85 and 0.88, demonstrating excellent stability.

#### 2.2.4 Self-compassion

Self-compassion was evaluated using the revised Self-Compassion Scale for Adolescents, based on Neff's original framework and adapted by Gong et al. (30). This instrument consists of 12 items distributed among three dimensions: self-kindness, common humanity, and mindfulness. Each item is rated on a 5-point scale ranging from 1 (almost never) to 5 (almost always). Higher scores on this scale indicate greater levels of self-compassion. The scale has demonstrated robust internal consistency, evidenced by a Cronbach's alpha of 0.770.

### 2.3 Data collection

#### 2.3.1 Diagnostic criteria

The diagnostic criteria for adolescents with PCOS in this study were based on the guidelines established by the American Society for Reproductive Medicine and the European Society of Human Reproduction and Embryology (31, 32). These guidelines include: (1) Ultrasonographic Evidence: The diagnosis requires the presence of polycystic ovaries, either through an ovarian volume exceeding 10 cm3 or by identifying at least 12 follicles with diameters ranging from 2 to 9 mm on one or both ovaries. (2) Clinical Evidence of Hyperandrogenism: Diagnosed through elevated blood androgen levels. (3) Menstrual History: A history of menstruation for at least 2 years with infrequent or absent periods is required.

For hyperandrogenism in this study, diagnosis was based on androgen levels measured in our laboratory and compared with established local norms for women of reproductive age (33). Hyperandrogenism was confirmed if dehydroepiandrosterone sulfate levels were above 8.71 mmol/L or testosterone levels exceeded 0.48 mg/L. The severity of acne was assessed using the Pillsbury classification system, with scores from 3 to 5 indicating moderate cystic acne, scores from 1 to 2 indicating mild acne, and a score of 0 indicating no acne (34). The presence of hirsutism was evaluated using the modified Ferriman-Gallwey (mF-G) scoring system, as recommended by the WHO, with a score >6 confirming hirsutism (35).

#### 2.3.2 Sample and procedure

Puberty is typically characterized as the period between the ages of 10 and 20 (36). This cross-sectional study was conducted on adolescents aged 15 to 20 years who had been diagnosed with PCOS. The diagnostic criteria for PCOS include having at least 2 years of menstruation and typically experiencing menarche between the ages of 12 and 16 years. The research was carried out at Shanghai First Maternity and Infant Hospital between January 2020 and December 2021. Prior to completing the questionnaire, all participants provided written informed consent. The studies involving human participants were reviewed and approved by the Ethics Committee of Shanghai First Maternity and Infant Hospital. The research adhered to the “Measures for Ethical Review of Biomedical Research Involving Human Beings (Trial)” issued by the Ministry of Health, as well as the relevant provisions of the Declaration of Helsinki pertaining to biomedical experiments on human subjects.

The study's inclusion criteria encompassed: (1) meeting the recognized diagnostic criteria for polycystic ovarian syndrome in teens. (2) Women aged 15 to 20. (3) Absence of any previous ovarian surgery within the last 6 months. (4) No administration of drugs that impact the endocrine system within the previous 3 months. (5) A minimum level of literacy at the elementary school level is required to ensure that the questionnaires can be completed without assistance. (6) After receiving comprehensive information on the study and have acquired explicit consent from the participant or their family.

The exclusion criteria encompassed: (1) The presence of diseases that result in heightened levels of androgens, such as diabetes mellitus, adrenal hyperplasia, Cushing's syndrome, or androgen-producing tumors. Additionally, other disorders that lead to ovulation disorders, such as hyperprolactinemia, premature ovarian failure, hypothalamic-pituitary amenorrhea, or thyroid dysfunction, are also considered exclusion criteria. (2) The inclusion of individuals with psychiatric problems that could potentially impact the study outcomes, insufficient clinical data, or participants who were lost to follow-up. (3) Congenital hypoplasia or malformation of the reproductive organs refers to a condition where the reproductive organs are underdeveloped or have structural abnormalities present from birth. (4) Severe problems affecting the kidneys, liver, heart, or blood. (5) Diseases that could impact the outcomes of the study, compromise the integrity of clinical data, or lead to loss of participants during the study period.

#### 2.3.3 Survey method

The required sample size for this study was calculated using the formula: n = ($Z_{a}^{2}\pi$(1-π))/δ^2^ (37). Based on literature, the estimated incidence of depression among individuals with PCOS is approximately 20% (38). Consequently, we used π = 20% for our sample size calculations. We set the allowable error, δ, at 5% to ensure adequate research precision. Given a 95% confidence interval with α = 0.05, the initial calculation required a sample size of 245 individuals. Anticipating a potential dropout rate of 20% (39), we adjusted the final sample size to 300 individuals to maintain statistical power.

The questionnaire for this study included sections on demographic information (such as age, height, and weight), along with measures for body dissatisfaction, self-esteem, self-compassion, and depression. We distributed a total of 300 questionnaires and received 287 valid responses, achieving a response rate of 95.7%. This high response rate indicates strong engagement and interest from the participants and adds robustness to the study findings.

### 2.4 Statistical analysis

Data were analyzed using IBM SPSS Statistics version 23.0 for Windows (IBMCorp., Armonk, NY, USA) and Andrew F. Hayes' PROCESS version 3.4.1 macro program's Model 4.

In this study, we first analyzed the general characteristics of participants. Continuous variables were presented as mean and standard deviation (M ± SD), and categorical variables displayed as frequency (n) and percentage (%). Secondly, correlation analysis was conducted to explore the relationships among body dissatisfaction, self-esteem, self-compassion, and depression.

Mediation analysis permits examination of process, allowing the research to investigate by what means X exerts its effect on Y (40). Thus, the third step, we used a parallel mediation model to assess the mediating roles of self-esteem and self-compassion on the relationship between body dissatisfaction and depression. The mediation method required the following conditions (Figure 1): (1) Body dissatisfaction was significantly associated with depression (total effect: c coefficient). (2) Body dissatisfaction was significantly with self-esteem (a1 coefficient) and self-compassion (a2 coefficient). (3) When controlling for body dissatisfaction, self-esteem (b1 coefficient) and self-compassion (b2 coefficient) was significantly with depression. (4) When controlling for self-esteem and self-compassion, the relationship between body dissatisfaction and depression was reduced (direct effect: *c*′coefficient). The proportions mediated were determined by dividing the indirect effect (a1^*^b1, a2^*^b2) by the total effect (c coefficient) (41). All tests utilized the bias-corrected percentile bootstrap method, repeated 5000 times, calculating 95% confidence intervals (CIs). Significance was determined if CIs did not include 0 (42). The level of statistical significance was set at *p* < 0.05.

## 3 Results

### 3.1 Participant characteristics

Table 1 summarizes the demographic characteristics of the study participants. On average, participants were 18 years old with a mean body mass index (BMI) of 21 kg/m^2^. Specifically, among the study group, 70 patients (24.39%) were reported to have acne, 56 (19.51%) had hirsutism, and another 56 (19.51%) exhibited acanthosis nigricans.

**Table 1:** Demographic characteristics for the study subjects (*N* = 287).

**Variables**	**Categories**	**Mean/*n***	**SD/%**
Age (years)		18	1.27
BMI (kg/m^2^)		21	3.02
Acne	Yes = 1	70	24.39
	No = 0	217	75.61
Hirsutism	Yes = 1	56	19.51
	No = 0	231	80.49
Acanthosis nigricans	Yes = 1	56	19.51
	No = 0	231	80.49

Table 2 provides the characteristics of body dissatisfaction, depression, self-esteem, and self-compassion among adolescents with PCOS. The average score for body dissatisfaction was 2.50 (standard deviation, SD = 0.71), with 73.52% of participants expressing dissatisfaction with their body image. In detail, the scores for satisfaction with overall appearance, fatness, and facial looks were 2.79 (SD = 0.83), 2.45 (SD = 0.89), and 2.41 (SD = 0.83) respectively. The average score for depression was 16.88 (SD = 10.70), indicating that 48.78% of participants showed symptoms of depression. Additionally, the average scores for self-esteem and self-compassion were 27.32 (SD = 4.67) and 39.26 (SD = 6.84), respectively.

**Table 2:** Descriptive statistics in body dissatisfaction, depression, self-esteem and self-compassion (*N* = 287).

**Variables**	**Mean ±SD**	**Range**	**Categories**	***n***	**%**
Body dissatisfaction	2.50 ± 0.71	[1.1, 4.6]	Satisfied = 0	76	26.48
			Dissatisfied = 1	211	73.52
Overall appearance	2.79 ± 0.83	[1.0, 5.0]	Satisfied = 0	36	12.54
			Dissatisfied = 1	251	87.46
Fatness	2.45 ± 0.89	[1.0, 5.0]	Satisfied = 0	97	33.80
			Dissatisfied = 1	190	66.20
Facial looks	2.41 ± 0.83	[1.0, 5.0]	Satisfied = 0	92	32.06
			Dissatisfied = 1	195	67.94
Depression	16.88 ± 10.70	[0.0, 47.0]	None [0–15]	147	51.22
			Possible [16–60]	140	48.78
Self-esteem	27.32 ± 4.67	[10.0, 38.0]			
Self-compassion	39.26 ± 6.84	[21.0, 57.0]			

### 3.2 Correlation analysis

Table 3 presents the correlation analysis results, exploring the relationships among body dissatisfaction, self-esteem, self-compassion, and depression. Body dissatisfaction showed a significant positive correlation with depression (*r* = 0.586, *p* < 0.001). In contrast, body dissatisfaction negatively correlated with self-esteem (*r* = −0.518, *p* < 0.001) and self-compassion (*r* = −0.474, *p* < 0.001). Moreover, depression also showed significant negative correlations with self-esteem (*r* = −0.699, *p* < 0.001) and self-compassion (*r* = −0.619, *p* < 0.001).

**Table 3:** Correlation matrix of body dissatisfaction, self-esteem, self-compassion and depression (*N* = 287).

	**Body dissatisfaction**	**Self-esteem**	**Self-compassion**
Self-esteem	−0.518^***^	—	—
Self-compassion	−0.474^***^	0.682^***^	—
Depression	0.586^***^	−0.699^***^	−0.619^***^

^***^p < 0.001. We controlled for age, BMI, acne, hirsutism, and acanthosis nigricans.

### 3.3 Mediation effects

Table 4 illustrates the results of a mediation analysis that controlled for age, BMI, acne, hirsutism, and acanthosis nigricans to explore the roles of self-esteem and self-compassion as mediators in the relationship between body dissatisfaction and depression. In Model 1 and Model 2, body dissatisfaction positively predicted depression (β = 9.184, *p* < 0.001) and negatively impact self-esteem (β = –3.608, *p* < 0.001) and self-compassion (β = −4.795, *p* < 0.001). In Model 3, which included all three variables simultaneously, showed that body dissatisfaction had a significant positive effect on depression (β = 4.254, *p* < 0.001). Self-esteem (β = −0.944, *p* < 0.001) and self-compassion (β = −0.318, *p* < 0.001) exhibited significant negative effects on depression. Model 3 explained 60.6% of the variance in depression (Adjusted *R*^2^ = 0.606, *F* = 55.961, *p* < 0.001).

**Table 4:** Result of mediating effect analysis (*N* = 287).

**Variables**	**Model 1**	**Model 2**		**Model 3**
	**Depression**	**Self-esteem**	**Self-compassion**	**Depression**
Age	0.263 (0.660)	0.030 (0.163)	−0.334 (−1.197)	0.186 (0.577)
BMI	−0.394^*^(−2.345)	0.235^**^(2.986)	0.204 (1.732)	−0.107 (−0.784)
Acne	−0.387 (−0.309)	−0.565 (−0.962)	1.081 (1.232)	−0.576 (−0.566)
Hirsutism	−2.054 (−1.408)	0.853 (1.248)	−0.673 (−0.659)	−1.463 (−1.238)
Acanthosis nigricans	−0.680 (−0.477)	0.550 (0.824)	1.669 (1.673)	0.369 (0.321)
Body dissatisfaction	9.184^***^(12.086)	−3.608^***^(−10.139)	−4.795^***^(−9.016)	4.254^***^(5.846)
Self-esteem				−0.944^***^(−7.498)
Self-compassion				−0.318^***^(−3.777)
R^2^	0.403	0.314	0.283	0.617
Adjusted R^2^	0.390	0.300	0.268	0.606
F	31.437^***^	21.283^***^	18.461^***^	55.961^***^

^*^p < 0.05, ^**^p < 0.01, ^***^p < 0.001. The t-value was presented in parentheses.

Table 5 provides the significance of the mediating effects of self-esteem and self-compassion. The total effect of body dissatisfaction on depression was statistically significant [β = 9.184, 95% CI = (7.695, 10.674)], supporting H1. The total indirect effect was also statistically significant [β = 4.930, 95% CI = (0.250, 0.402)], with the contributions of self-esteem [β = 3.405, 95% CI = (0.151, 0.305)] and self-compassion being [β = 1.525, 95% CI = (0.045, 0.165)], supporting H2 and H3. These mediation effects accounted for 37.07% and 16.61% of the total effect, respectively.

**Table 5:** Significance test of mediating effects (*N* = 287).

**Model**	**β**	**Boot SE**	**95%CI**	**Percent mediation (%)**
Direct effect	4.254^***^	0.728	2.828, 5.681	
Total indirect effects	4.930^***^	0.039	0.250, 0.402	
Indirect effect 1	3.405^***^	0.039	0.151, 0.305	37.07%
Indirect effect 2	1.525^***^	0.031	0.045, 0.165	16.61%
Total effect	9.184^***^	0.760	7.695, 10.674	

^***^p < 0.001.

## 4 Discussion

To our knowledge, this is the first study to use mediation analysis to explore the role of self-esteem and self-compassion in the relationship between body dissatisfaction and depression among adolescent with PCOS. Mediation modeling is crucial as it helps to elucidate the underlying mechanisms through which body dissatisfaction influences depression. By identifying self-esteem and self-compassion as key mediators, our study provides a deeper understanding of these psychological processes, which is pivotal for developing effective prevention and intervention strategies aimed at improving mental health outcomes for PCOS patients.

Our results identified a significant positive correlation between body dissatisfaction and depression in adolescents with PCOS, aligning with the findings of Paxton et al. (43). Specifically, the study indicated that higher levels of dissatisfaction with body appearance correlated with more pronounced depressive symptoms. Body dissatisfaction typically arises from a significant disparity between one's expected and actual appearance. Societal beauty standards, often extensively promoted by the media, such as idealizing thinness and beauty, exert substantial pressure on individuals. This study showed that the average self-evaluation scores for body fatness and facial looks in adolescents with PCOS were above 2 points, reflecting their dissatisfaction with their body shape and looks. Furthermore, individuals often compare themselves to others; however, when such comparisons are based on unrealistic standards, they usually lead to reduced body satisfaction, feelings of inferiority, and negative emotions (44). Previous research has shown that about 60% of adolescents report body dissatisfaction (45), with adolescent girls particularly exhibiting lower levels of body satisfaction (46). Moreover, adolescents with PCOS typically exhibit even lower body satisfaction levels compared to their healthy counterparts (9, 11). Depression can stem from various sources, including low mood and negative life events (47). As body dissatisfaction is a manifestation of negative emotions, higher levels of body dissatisfaction increase the likelihood of experiencing depression. Numerous studies with adolescent girls have demonstrated that body dissatisfaction can positively predict the onset of depression (48, 49). These studies emphasize the importance of promoting an objective, realistic, and positive body image among adolescents to support their mental health. Therefore, it is essential to implement prevention and intervention measures to reduce the risk of depression.

The results of mediation analysis showed that self-esteem partially mediated the relationship between body dissatisfaction and depression, accounting for 37.07% of the total effect. This aligns with the research of Jiang et al. and Bazarganipour et al. (9, 50). Specifically, higher levels of body dissatisfaction were associated with lower self-esteem and more pronounced depressive symptoms. Previous research has shown that negative evaluations of one's body can reduce self-esteem in adolescents, thereby affecting their physical and mental health development (51). This is particularly relevant during adolescence, a period when individuals undergo significant physical changes and become increasingly conscious of their social appearance. For those with PCOS, symptoms like obesity, acne, hirsutism, and acanthosis nigricans can adversely affect their body image, leading to increased social ridicule, rejection, and diminished life satisfaction, which in turn impact their self-esteem and contribute to the development of depression (52). These findings support the vulnerability-stress model of depression, which suggests that not all individuals with body dissatisfaction will develop severe depression. The likelihood of depression depends on other factors, such as self-esteem and attribution style. Those with lower levels of these qualities may require less stress to trigger depressive symptoms (53). Therefore, to improve self-esteem in PCOS patients, interventions can start by helping patients identify and change negative thought patterns and behaviors through cognitive-behavioral therapy (CBT), thereby fostering more positive self-perception. Additionally, promoting a healthy lifestyle through balanced nutrition and regular exercise can enhance body image, further boosting self-esteem. Finally, strengthening social support for PCOS patients by providing education on PCOS and its psychological impacts can increase patients' understanding and self-management abilities, reduce misunderstandings and fears, and ultimately enhance self-efficacy and self-esteem.

Moreover, the study revealed that self-compassion also played a partial mediating role in the relationship between body dissatisfaction and depression, accounting for 16.61% of the total effect. This finding is consistent with the research by Liu and Zhang and Hua (54, 55). Specifically, higher levels of body dissatisfaction were linked to lower self-compassion and more pronounced depressive symptoms. Previous studies have suggested that body dissatisfaction can indirectly predict depression through the mediating role of self-compassion (55). In contexts such as breast cancer, self-compassion has been found to mediate the relationship between body image and distress (56). The mediating effect of self-compassion is based on the critical self-perception associated with body dissatisfaction, while self-compassion promotes a more accepting and kind attitude toward oneself. This approach helps adolescents with PCOS adopt a more inclusive perspective, reducing excessive focus on or identification with disliked body images and preventing deep immersion in distress. As a result, it alleviates body dissatisfaction, body shame, and ultimately reduces the occurrence of depression (53). Individuals with high levels of self-compassion are more likely to resist the influence of media promoting an “ideal thinness” culture and negative evaluations or ridicule about their body image. They are better equipped to maintain a positive evaluation of their physical appearance and experience fewer negative emotions (57). Therefore, it is vital to enhance the self-compassion levels of adolescents with PCOS patients. Firstly, self-compassion training through practices such as self-care exercises and mindfulness meditation can help patients be kinder to themselves in the face of difficulties, reduce self-criticism, enhance psychological resilience, and improve emotional regulation, thereby increasing self-compassion. Secondly, improving public understanding and acceptance of PCOS, providing more mental health resources and support services, and enhancing the support from family and friends can offer greater care and assistance to PCOS patients. This can reduce their feelings of shame and improve overall quality of life.

This study also discovered that both self-esteem and self-compassion play crucial roles in mediating the relationship between body dissatisfaction and depression. While self-esteem had a slightly stronger mediating effect, both factors are indispensable, consistent with the findings of Zhu et al. (58). Self-esteem and self-compassion are personality traits that significantly contribute to reducing depression and fostering a positive outlook on life. Self-esteem often relies on comparisons with others, while self-compassion highlights the importance of self-satisfaction without the need for comparison. These two traits are inherently correlated, with individuals possessing high self-compassion also tending to have high self-esteem (59, 60). Therefore, addressing both physical symptoms of PCOS and psychological aspects like self-esteem and self-compassion can lead to more comprehensive care. Healthcare providers can develop multifaceted treatment plans that include psychological support alongside medical treatment for PCOS. Schools and community programs can incorporate activities and workshops to promote positive body image and mental health resilience. Policy-makers can advocate for the inclusion of psychological wellbeing measures in routine care for adolescents with PCOS.

This study had some limitations. Firstly, it focused on subjective cognitive factors such as self-esteem and self-compassion as mediators between body dissatisfaction and depression. Other factors, such as social support and mass media, also impact body satisfaction and depression. Future studies will explore the role of these additional factors in the relationship between body dissatisfaction and depression. Secondly, this study specifically analyzed adolescents with PCOS and did not include a control group of non-PCOS individuals of the same age. Future studies will incorporate a control group to provide supplementary evidence. Finally, In China, a unified consensus on diagnostic criteria for adolescents with PCOS has not yet been established. Therefore, this study utilized the criteria based on Shanghai First Maternity and Infant Hospital. Future studies will continue to be strengthened to enhance the accuracy of diagnosing adolescents with PCOS.

## 5 Conclusion

This study examined the roles of self-esteem and self-compassion in the relationship between body dissatisfaction and depression among adolescents with PCOS. The findings revealed that self-esteem and self-compassion partially mediated this relationship. The clinical significance of this study lies in its potential to inform better mental health support strategies for adolescents with PCOS. By identifying self-esteem and self-compassion as mediating factors, healthcare providers can develop targeted interventions that not only address body dissatisfaction but also enhance overall mental health, thereby reducing the risk of depression in this vulnerable population.

## Data availability statement

The raw data supporting the conclusions of this article will be made available by the authors, without undue reservation.

## Ethics statement

The studies involving humans were approved by the Ethics Committee of Shanghai First Maternity and Infant Hospital (Approval No.: KS22219). The studies were conducted in accordance with the local legislation and institutional requirements. The participants and the participants' legal guardians/next of kin provided their written informed consent to participate in this study.

## Author contributions

HH: Data curation, Formal analysis, Funding acquisition, Methodology, Software, Validation, Writing – original draft, Writing – review & editing. LL: Conceptualization, Data curation, Funding acquisition, Project administration, Supervision, Validation, Writing – original draft, Writing – review & editing. WS: Investigation, Project administration, Resources, Supervision, Writing – original draft, Writing – review & editing.
